# Combining human liver ECM with topographically featured electrospun scaffolds for engineering hepatic microenvironment

**DOI:** 10.1038/s41598-024-73827-5

**Published:** 2024-10-05

**Authors:** Yunxi Gao, Victoria L. Gadd, Maria Heim, Rhiannon Grant, Thomas S. R. Bate, Hannah Esser, Sofia Ferreira Gonzalez, Tak Yung Man, Stuart J. Forbes, Anthony Callanan

**Affiliations:** 1 Institute for Bioengineering, School of Engineering, University of Edinburgh, Edinburgh, UK; 2 Centre for Regenerative Medicine, Institute for Regeneration and Repair, University of Edinburgh, Edinburgh, UK; 3 MERLN Institute for Technology-Inspired Regenerative Medicine, Maastricht University, Maastricht, The Netherlands; 4 Department of Visceral, Transplant and Thoracic Surgery, Medical University of Innsbruck, Innsbruck, Austria; 5 Centre for Inflammation Research, Institute for Regeneration and Repair, University of Edinburgh, Edinburgh, UK; 6 Vanderbilt University Medical Center, Nashville, USA; 7 Foundation of Liver Research, The Roger Williams Institute of Liver Study, London, UK

**Keywords:** Electrospinning, Cell scaffold, Liver tissue engineering, Topography, Decellularization, Human extracellular matrix, Biological models, Nanofabrication and nanopatterning, Nanopores, Biomaterials, Cell delivery, Tissue engineering, Biological techniques, Biotechnology, Engineering, Biomedical engineering, Bioinspired materials, Materials science, Biomaterials, Nanoscience and technology, Nanobiotechnology, Nanofabrication and nanopatterning, Nanopores, Nanostructures

## Abstract

Liver disease cases are rapidly expanding worldwide, and transplantation remains the only effective cure for end-stage disease. There is an increasing demand for developing potential drug treatments, and regenerative therapies using in-vitro culture platforms. Human decellularized extracellular matrix (dECM) is an appealing alternative to conventional animal tissues as it contains human-specific proteins and can serve as scaffolding materials. Herein we exploit this with human donor tissue from discarded liver which was not suitable for transplant using a synergistic approach to combining biological and topographical cues in electrospun materials as an in-vitro culture platform. To realise this, we developed a methodology for incorporating human liver dECM into electrospun polycaprolactone (PCL) fibres with surface nanotopographies (230–580 nm). The hybrid scaffolds were fabricated using varying concentrations of dECM; their morphology, mechanical properties, hydrophilicity and stability were analysed. The scaffolds were validated using HepG2 and primary mouse hepatocytes, with subsequent results indicating that the modified scaffolds-maintained cell growth and influenced cell attachment, proliferation and hepatic-related gene expression. This work demonstrates a novel approach to harvesting the potential from decellularized human tissues in the form of innovative in-vitro culture platforms for liver.

**Supplementary Information:**

The online version contains supplementary material available at 10.1038/s41598-024-73827-5.

## Introduction

A Lancet commission recently reported that the mortality due to liver disease has risen 400% since 1970, accounting for 2 million deaths per year^1^. The Office for National Statistics stated that in 2020, liver cirrhosis and its related diseases became the second most common cause of premature death in the UK^2^. Currently, the only curative treatment is liver transplantation. However, transplantation possesses several limitations, including the severe shortage of donor livers, increasing length of waiting lists, and possible post-transplant complications^3^. Furthermore, the emergence of the novel respiratory virus COVID-19 has dramatically disrupted transplantations worldwide^4^. Tissue engineering has provided an alternative pathway to donor livers. The last decade’s progress on engineered constructs has shown promising opportunities for novel treatments of liver disease^5–7^. Tissue-like scaffolds not only serve as cell carriers but also provide a growth environment for cellular communication and function maintenance. Natural polymers for example collagen, gelatin, alginate, chitosan and fibrin, synthetic polymers such as poly lactic acid (PLA), polyglycolic acid (PGA), Polycaprolactone (PCL), and the combination of them, are widely used as matrix biomaterials due to their high biocompatibility and mechanical performance for hepatocytes growth^8–11^. Techniques including whole organ decellularization^12^, electrospinning^10^, 3D printing^13^, and freeze-drying^14^are commonly used to produce 3D constructs, which can mimic the liver structure and composition. Microfluidic devices known as liver-on-a chip, can precisely control fluid flows at micro-scale and drive multiple cell types to recapitulate the physiological functions of tissue^15^. Moreover, 3D bioprinting can fuse cells and growth factors in bioink to construct liver microtissues with precise microstructures showing promise in tissue regeneration^6^.

The in vivo extracellular matrix (ECM) provides biophysical cues including surface topography, matrix stiffness and mechanical force, that cells interact with^16^. Each tissue has its own distinct ECM composition and topography that interacts dynamically with its various cellular components^17^. Electrospinning is one of the most commonly used techniques for the development of fibrous ECM mimicking scaffolds. It is widely used in all fields of tissue engineering due to the ease of manipulation of properties such as pore size, porosity, fibre diameter, orientation and nano-scale topographies^18–22^. Nano-scale topographies and patterns on polymer substrates are critical features that drive phenotypic behaviours for many types of cells^14,16,23,24^. Vasudevan et al. recently reviewed the progress of electrospinning for liver tissue engineering and crystallised that, in multiple studies, architecture and composition of the substrates have a direct influence on cell function^25^. Therefore, en route to a tissue engineered solution for the donor liver shortage it is crucial to replicate the biophysical structure of liver ECM, using biomaterial-based approaches to investigate its impact on hepatocytes.

Previous studies, including those from our group, have shown that liver cells respond to changes in morphology (fibre size, orientation, and porosity), topography (micro/nano topography) and composition (ECM proteins) of electrospun scaffolds^8,10,12,18,26–29^. To date however, research regarding synergistic effects of biochemical and biophysical cues has mainly focused on macroscopic topography and cell types other than hepatocytes^30,31^. Recent advances in manufacturing allow us to interrogate nano-micro topographies. Non-solvent-induced phase separation (NIPS) is a common mechanism used to initiate porous structures on a polymeric material^32^. A ternary system contains a mixture of a highly volatile solvent, less volatile solvent with polymer, and the difference in evaporation rate generates phase separation in this system^33^. Porous fibre structures can also be induced by vapour-induced phase separation (VIPS), where water vapour present in the air lead to phase separation resulting porous in the bulk of the fibres^34^.

In the past decade, liver tissue engineers have made enormous developments in liver scaffolds with complex architectures and compositions to manipulate hepatocyte behaviour and function^7,25^. In vivo, cells attach to ECM through interactions between membrane bound integrins and ECM adhesion motifs (such as RGD (collagen, fibronectin), and YIGSR (laminin))^35,36^. However, conventional synthetic polymer materials lack these adhesion sites^37^. To mediate this lack of adhesion motifs, researchers modify biomaterial substrates with bioactive molecules, such as ECM proteins, growth factors and decellularized extracellular matrix (dECM)^8,9,38^ Signals from the ECM in the hepatic microenvironment display a convoluted interplay^39^. Due to their complex influence on cells, extensive research has been conducted on hepatic dECM sourced from various mammals, including rats, pigs, sheep and humans, both by incorporating the ECM into biomaterial scaffolds, and decellularizing whole organs for subsequent recellularization with healthy hepatocytes^9,13,40–44^. Previous studies including ours, have shown that integrating rodent or human dECM into electrospun scaffolds aids in maintaining liver-specific functions to a greater extent than individual ECM proteins^45,46^.

**Fig. 1:**
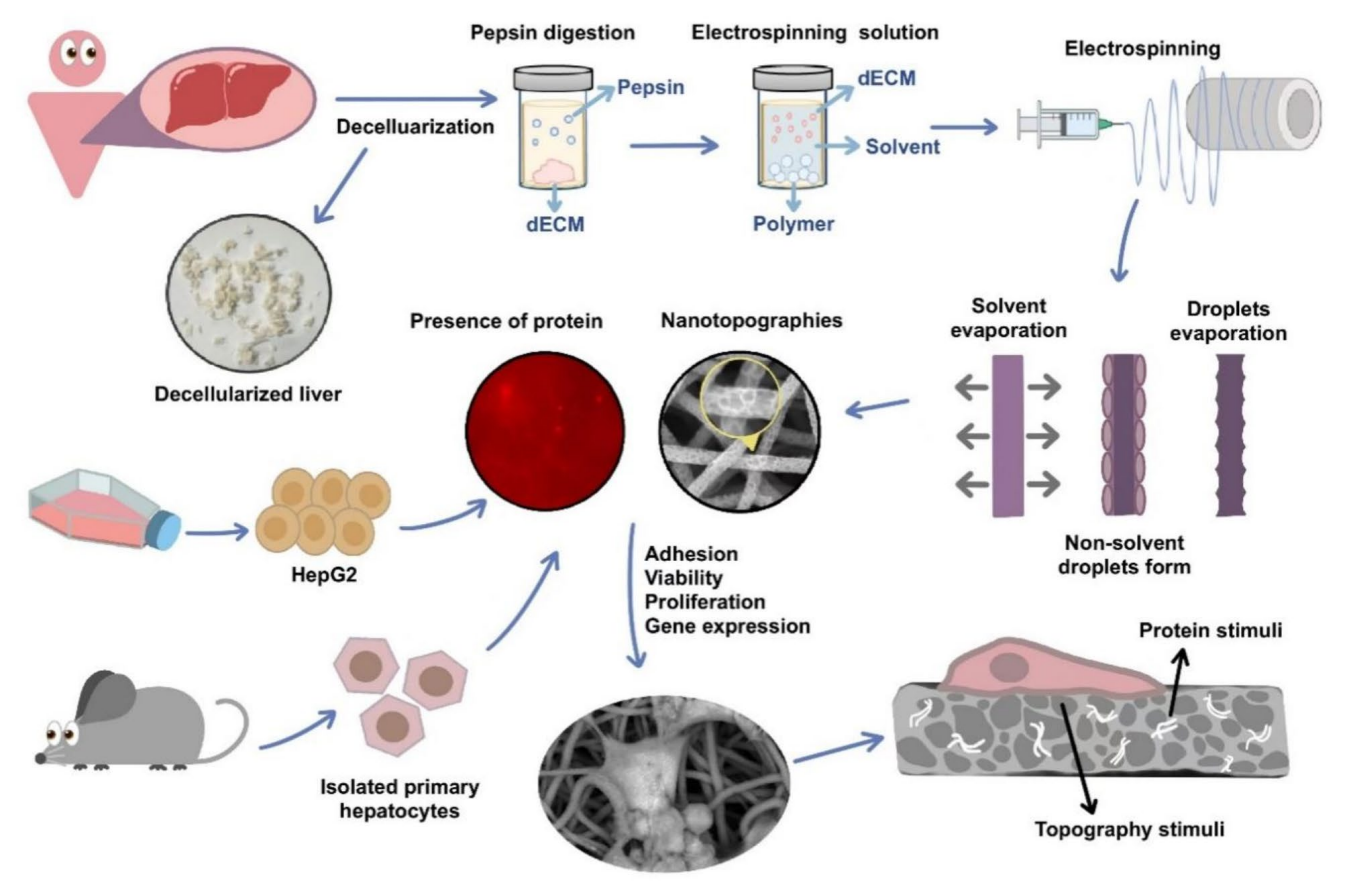
Schematic representation of the study.

It is well-established that the matrisome expression patterns are distinct between species, age, sex, tissue and disease progression^39,47,48^. Human derived-ECM could provide lower immunogenicity than animal tissue and could preserve human-specific proteins^48^. We therefore sought to repurpose discarded human tissue, unsuitable for transplant, as a biomaterial for hepatic tissue engineering. In this study, a novel bioactive hybrid scaffold was developed with topographically modified PCL fibres combined with human liver dECM, in attempt to create a customizable ECM microenvironment. Our previous studies demonstrated that nanotopography promotes the proliferation of hepatocytes, showed manufacturability and the influences with the incorporation of rat liver dECM^18,28^. This study is the first of its kind which incorporated human dECM directly into electrospun fibres with surface nanotopographies, without requiring post-treatment or additional chemicals (Fig. 1). HepG2 cells and mouse primary hepatocytes (MPHs) were used to validate these scaffolds. HepG2 cells are widely used in in vitro liver model studies due to their phenotypic stability^49^. Additionally, they act as excellent benchmarking cells for material optimisation, but don’t truly reflect the primary hepatocyte function. Thus, MPHs were also chosen to provide direct insight into hepatocytes’ interactions with environmental factors. Mechanical testing revealed consistent morphology across the scaffolds in spite of altering the topography and composition. Cell analyses, including viability, proliferation, morphological assays, albumin secretion, and key hepatic gene expression confirmed the great potential of this approach in liver tissue engineering.

## Results

### Tissue decellularization

**Fig. 2:**
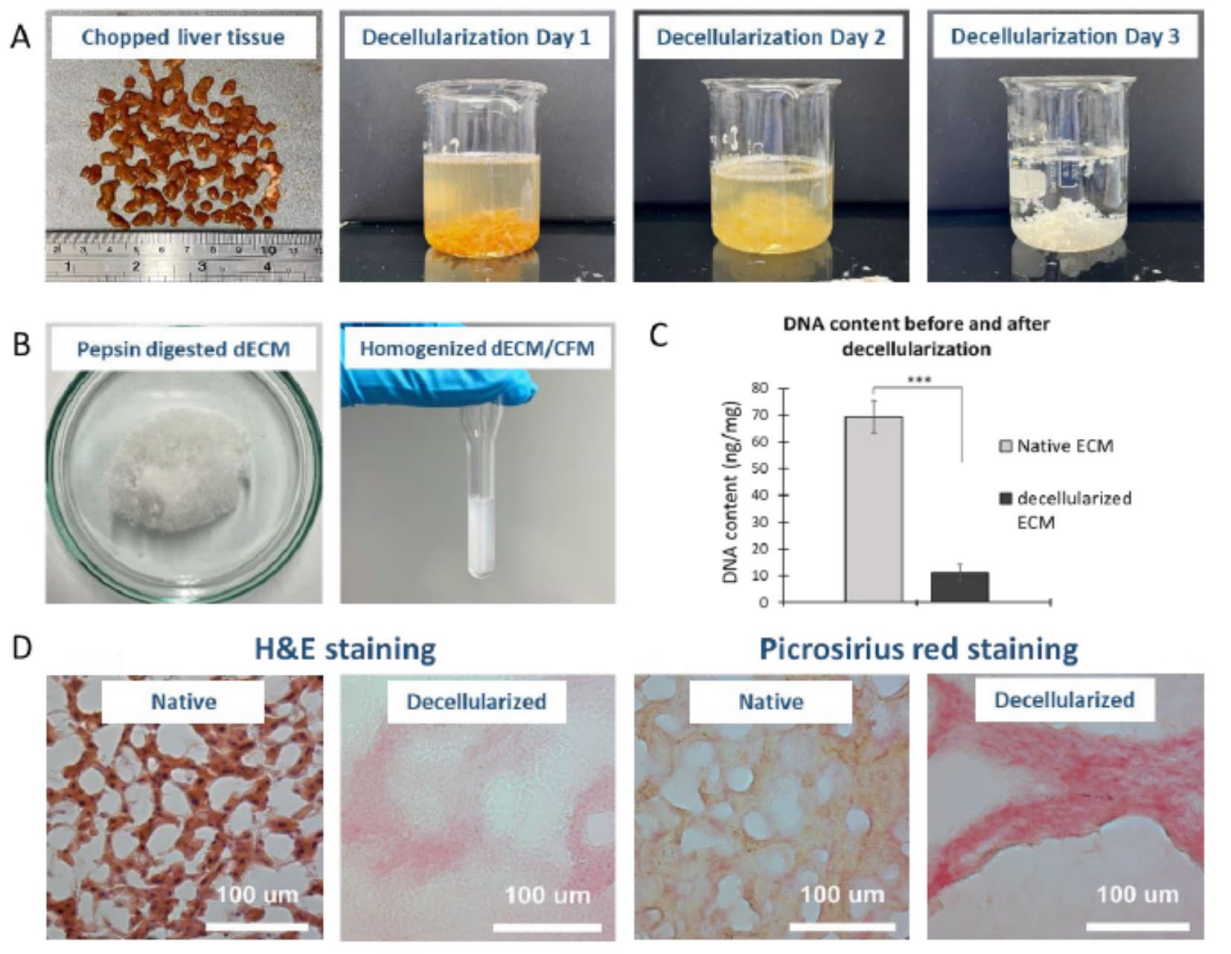
(**A**) Chopped human liver tissue faded during decellularization; (**B**) Pepsin digested and homogenized dECM solution; (**C**) DNA content of liver tissue before and after decellularization, ***= P value < 0.001; (**D**) H&E (black = cell nuclei, light pink = ECM component) and Picrosirius red staining (yellow = nuclear component, deep pink = collagenous component) of liver tissue before and after decellularization, x20 magnification. One-way ANOVA results with Tukey’s Post-hoc test shown. ***= P value < 0.001. *N* = 3.

The human liver tissue was successfully decellularized, and a disc image of the tissue is shown in supplementary Fig. 2. Figure 2A depicts the colour of the tissue paled from red-brown to white. Pepsin digestion of the dECM resulted in a fine fibrous structure (Fig. 2B) after lyophilization. The DNA content of the tissue significantly dropped (*P* < 0.001, Fig. 2C). H&E and Picrosirius red staining of liver tissue before and after decellularization (Fig. 2D) demonstrate loss of cellular material. H&E staining confirmed the nuclear components had been removed; Picrosirius red staining demonstrated the preservation of the collagenous composition after decellularization.

### Scaffold properties

**Fig. 3:**
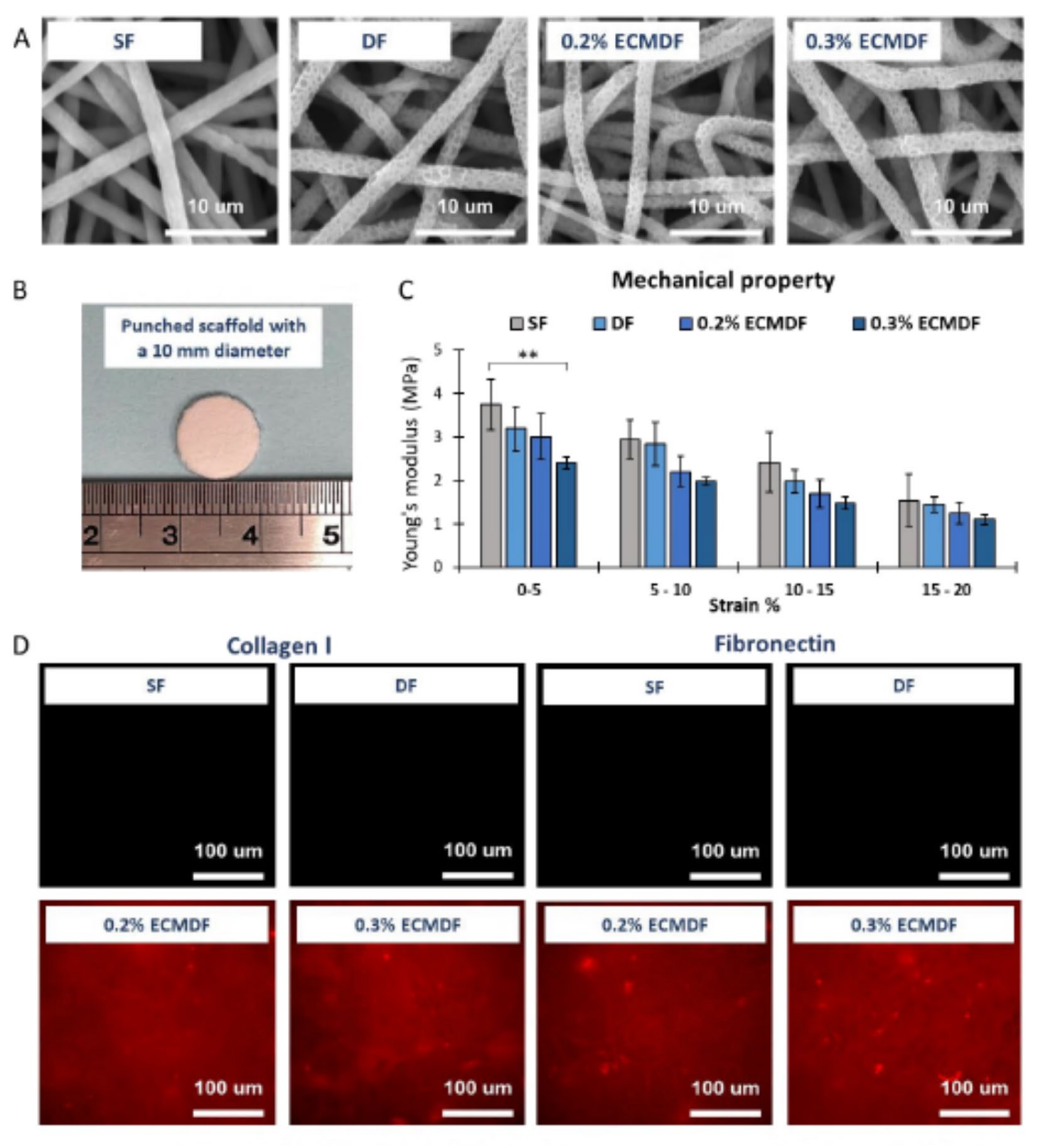
(**A**) SEM images of all types of scaffolds; (**B**) Image of the punched scaffold; (**C**) Young’s modulus of each type of scaffolds at different strain bands; (**D**) Immunostaining of collagen I and fibronectin on each type of scaffold, x40 magnification objective. One-way ANOVA results with Tukey’s Post-hoc test shown. **= P value < 0.01. *N* = 5.

Four different scaffolds were fabricated (Fig. 3A): smooth fibre scaffold (SF), depression fibre scaffold (DF), depression fibre scaffold with 0.2% dECM (0.2% ECMDF), depression fibre scaffold with 0.3% dECM (0.3% ECMDF). The depression was observed on the surface and outer edge of the fibre, with measurements indicating a depth of 0.25 μm. Similar notches of varying sizes are distributed on all fibre surfaces, confirming the porous structure of the depression fibre scaffold (see Supplementary Fig. 2). The fibres in all conditions had similar fibre diameters, with an average size of 2.25 μm (Table 1), and random morphology without alignment. The smallest fibre size was seen in 0.2% ECMDF at 1.84 μm and the largest fibre size was seen in DF at 2.73 μm. The largest variation was seen in 0.3% ECMDF with a difference of 0.36 μm between fibres. The depression diameters ranged from 230 to 580 nm, and there is no significant difference between groups. The average depression diameter is 0.4 ± 0.15 μm, calculated across all types of depression fibres. Figure 3B shows the morphology of a punched scaffold with a 10 mm diameter. Figure 3C shows Young’s modulus of each scaffold from tensile testing, a reduction gradient was noted between groups, and significance was observed between SF and 0.3% ECMDF. SF had the highest Young’s modulus in the 0–5% strain range (3.75 ± 0.24 MPa), followed by DFS (3.19 ± 0.17 MPa), 0.2% ECMDF (3 ± 0.05 MPa), and 0.3% ECMDF (2.4 ± 0.15 MPa). The collagen I and fibronectin staining confirmed the presence of dECM on the scaffolds (Fig. 3D). Contact angle measurements showed all scaffolds had a similar contact angle with an average value of 123.78° after 1 s (Table 1).

**Table 1:** Mechanical properties of each scaffold.

	SF	DF	0.2% ECMDF	0.3% ECMDF
Fibre diameter (µm)	2.14 ± 0.23	2.41 ± 0.32	2.16 ± 0.32	2.30 ± 0.36
Pore diameter (µm)	-	0.42 ± 0.16	0.43 ± 0.15	0.36 ± 0.13
Contact angle at 1s (°)	125.40 ± 2.26	122.30 ± 2.91	124.72 ± 2.95	122.70 ± 2.02

### In vitro degradation study

**Fig. 4:**
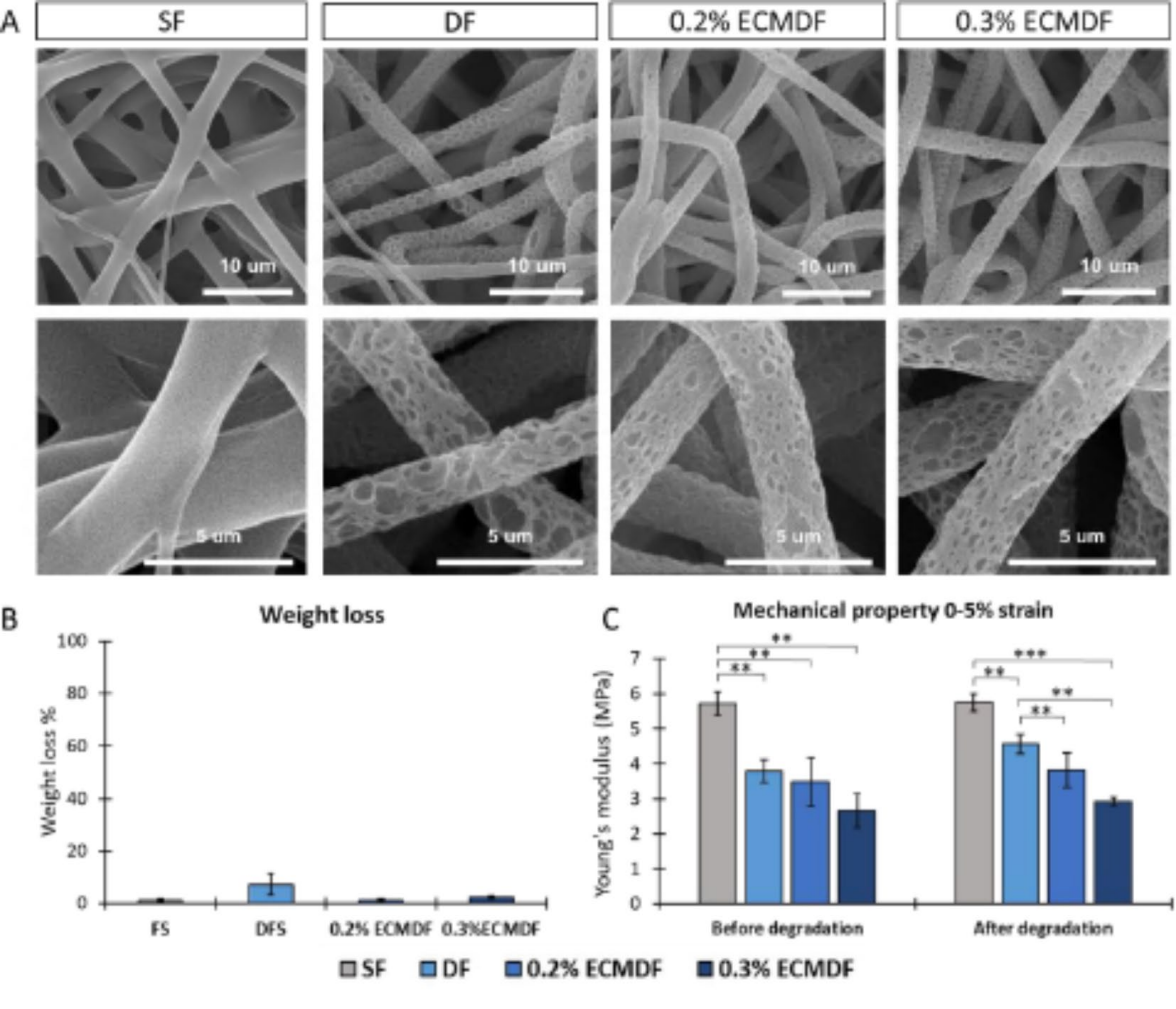
(**A**) SEM images of all types of scaffolds after degradation; (**B**) Weight loss percentage of each type of scaffold; (**C**) Young’s modulus of each type of scaffold at 0–5% strain band; One-way ANOVA results with Tukey’s Post-hoc test shown. ***= P value < 0.001. **= P value < 0.01. *= P value < 0.05. *N* = 5.

A hydrolytic degradation study was conducted to analyse the stability of the fibre topography, weight loss and mechanical properties over time in the culture media. As shown in Fig. 4A, there is no significant change in the topography of fibres after 14 days of incubation at 37 °C. The weight loss percentage (%) results (Fig. 4B) showed no significant difference between each scaffold, however, DFS showed a slightly higher average weight loss % compared to others. As shown in Fig. 4C, Young’s modulus of scaffolds has no significant change after degradation, but a slight increase was noted in all DF groups.

### Cell metabolic activity and proliferation

**Fig. 5:**
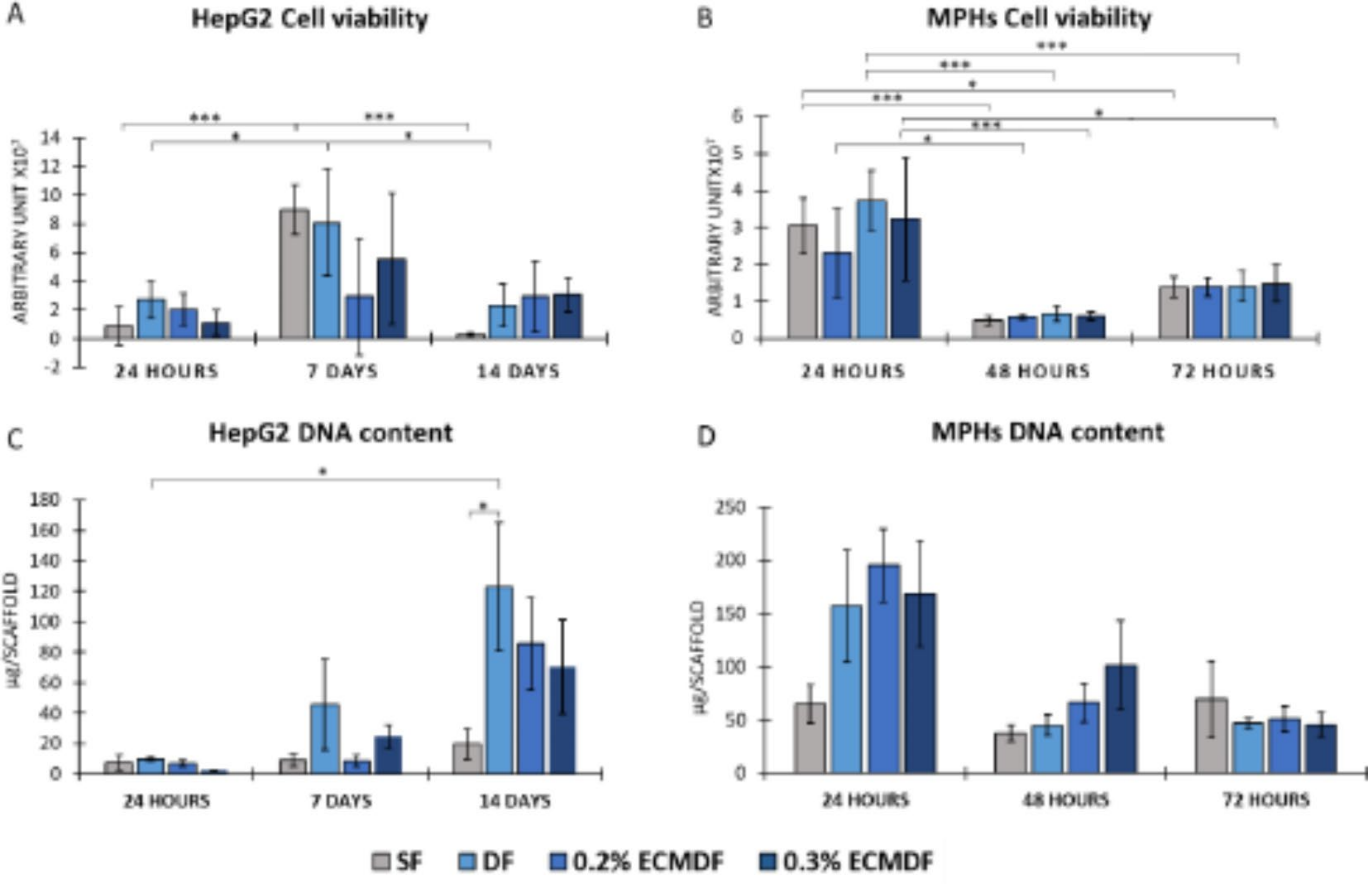
Cell analysis of different assays: (**A**) Cell viability of HepG2; (**B**) Cell viability of MPHs; (**C**) DNA quantification of HepG2; (**D**) DNA quantification of MPHs. One-way ANOVA results with Tukey’s Post-hoc test shown. ***= P value < 0.001, *= P value < 0.05. *N* = 5.

The CellTiter-Blue assay was conducted to determine the cell metabolic activity. The results in Fig. 5A showed a significantly increased fluorescence level of HepG2 on day 7 compared to 24 h, which decreased on day 14. The average fluorescence level showed the greatest reduction (97%) for SF between day 7 and day 14; DF and 0.3% ECMDF showed 71% and 45% reduction, respectively; however, 0.2% ECMDF displayed an increase in metabolic activity by 1% between day 7 and day 14. At each time point, all modified groups had a similar average fluorescence level, which was notably higher than SF on day 14. Statistically significant difference was not observed between the scaffolds but between time points. DNA quantification (Fig. 5C) of HepG2 cultured on modified scaffolds showed a consistent proliferation over the whole culture period. On day 14, the average DNA content of DF, 0.2% ECMDF and 0.3% ECMDF increased by 63%, 90% and 65%, respectively, compared to day 7. This validates the cell viability results, with all modified groups showing persistent metabolic and proliferative activities.

Figure 5B and D showed cell viability and DNA content for MPHs, which underwent a shorter culture period. In-line with HepG2, statistically significant differences were found between time points but not scaffolds. The highest cell viability was observed on all types of scaffolds at 24 h, followed by a reduction of 80% on average at 48 h. However, a recovery was observed at 72 h, with an average increase of 59% across all groups. A similar trend was observed for the DNA quantification results, which was highest at 24 h. Interestingly, the three modified scaffolds showed a higher DNA content compared to SF at 24 h, with the values for DF, 0.2% ECMDF and 0.3% ECMDF being 2.4, 3 and 2.6 times higher than that of SF. The fluorescence of all groups reduced by an average of 62% at 48 h, with the highest value observed for 0.3% ECMDF. The average DNA content at 48 and 72 h were very similar, at 44% and 45%, respectively. No significant differences were observed between groups and time points.

### Albumin secretion

**Fig. 6:**
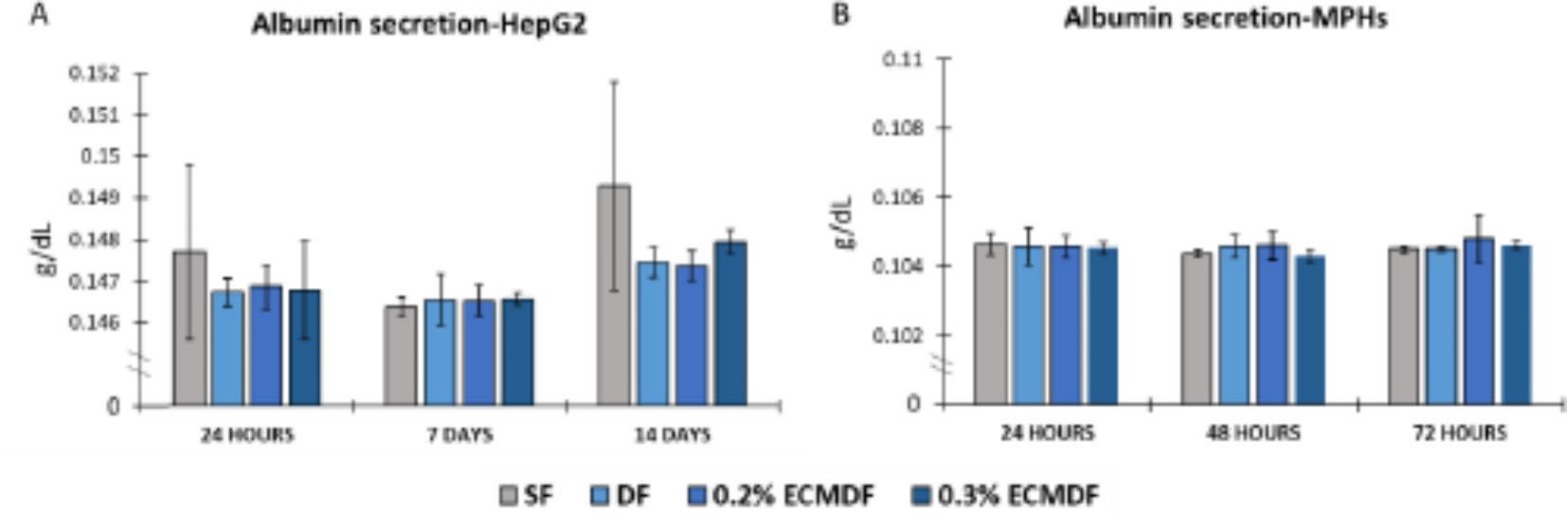
Albumin concentration in the culture media after 24 h of (**A**) HepG2 and (**B**) MPHs culture at each time point. *N* = 5.

Albumin concentration within the cell culture media over a 24 h period at all time points is displayed in Fig. 6. HepG2 secreted an average level of 0.147 g/dL, with a slight increase in the average value from 0.1465 g/dL at 7 days to 0.148 g/dL at 14 days. MPHs secreted an average level of 0.105 g/dL, showing no significant differences across groups and time points.

### Cell morphology and distribution on the scaffolds

**Fig. 7:**
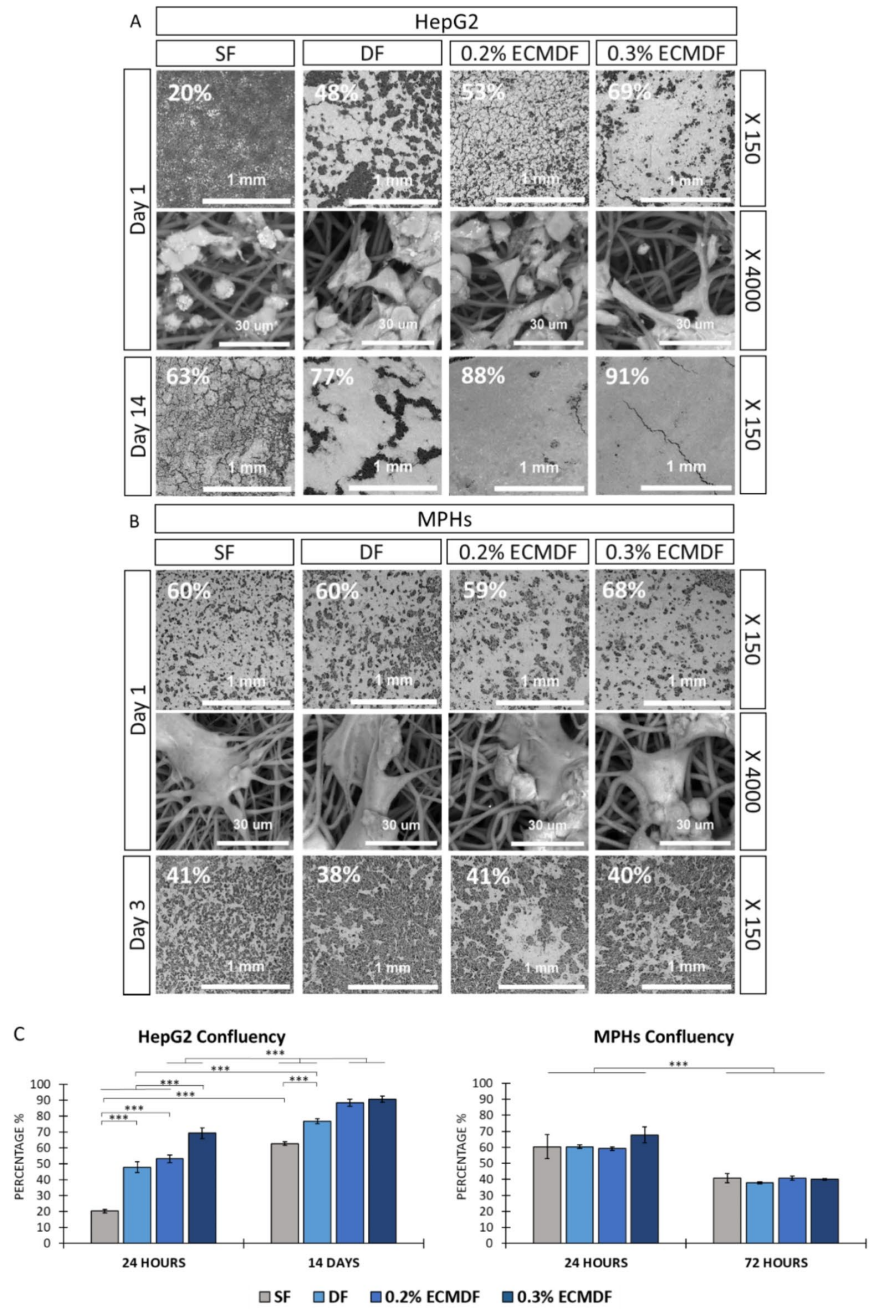
SEM images of osmium-stained adherent cells on all types of scaffolds at small (x150, scale bar = 1 mm) and large (x4000, scale bar = 30 μm) magnifications: (**A**) adherent HepG2 cells at 24 h and day 14; (**B**) adherent MPHs at 24 h and 72 h. The percentage numbers represent the average cell confluency; (**C**) cell confluency for HepG2 and MPHs at different time points. One-way ANOVA results with Tukey’s Post-hoc test shown. ***= P value < 0.001. *N* = 3.

Representative SEM images of HepG2 cultured on all scaffolds on day 1 and day 14 show the cell attachment, morphology, distribution and confluency (Fig. 7A). The cells cultured on SF presented a round globular shape on day 1, compared with those cultured on the other scaffolds, which all showed stretched and elongated shapes. On day 1, 0.3% ECMDF had the highest cell confluence percentage at 69 ± 3.4%, SF had the lowest at 20 ± 1.1%, and DF and 0.2% ECMDF had a similar at 48 ± 3.4% and 53 ± 2.4% respectively. Interestingly, the cell reached highly confluent on both 0.2% ECMDF and 0.3% ECMDF scaffolds on Day 14, with the values of 88 ± 2.3% and 91 ± 2%, which tended to form a cell monolayer-like structure, compared with 63 ± 3.2% for SF and 77 ± 1.5% for DF. Statistical significance was found between groups and time points (Fig. 7C).

Representative SEM images of MPHs cultured on all scaffolds showed very similar morphology and confluency between groups (Fig. 7B). The cells appeared to be elongated on all scaffolds on day 1, with 0.3% ECMDF having the highest cell confluence rate at 68 ± 5% and the remaining scaffolds having similar rates at an average of 60 ± 3.2%. However, the cell confluency decreased to an average of 40 ± 1.3% on all scaffolds on day 3. Statistical significance was found between time points (Fig. 7C).

**Fig. 8:**
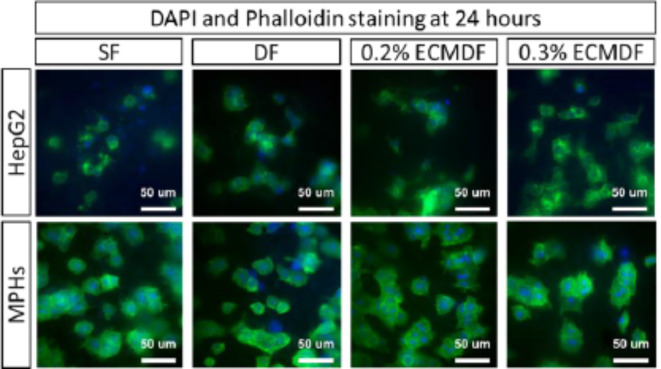
DAPI (blue = nuclei) and Phalloidin (green = actin filament) staining of HepG2 and MPHs cultured on each type of scaffolds for 24 h, images were taken with 40X objective, scale bar = 50 μm.

Representative fluorescent staining images are shown in Fig. 8. The morphology of attached HepG2 and MPHs can be observed after 24 h of culture. Both single and connected cells were seen across all scaffolds and both types of cells. More protrusions from the cells can be seen on the MPHs images which represent more attachments to surrounding fibres.

### Gene expression

**Fig. 9:**
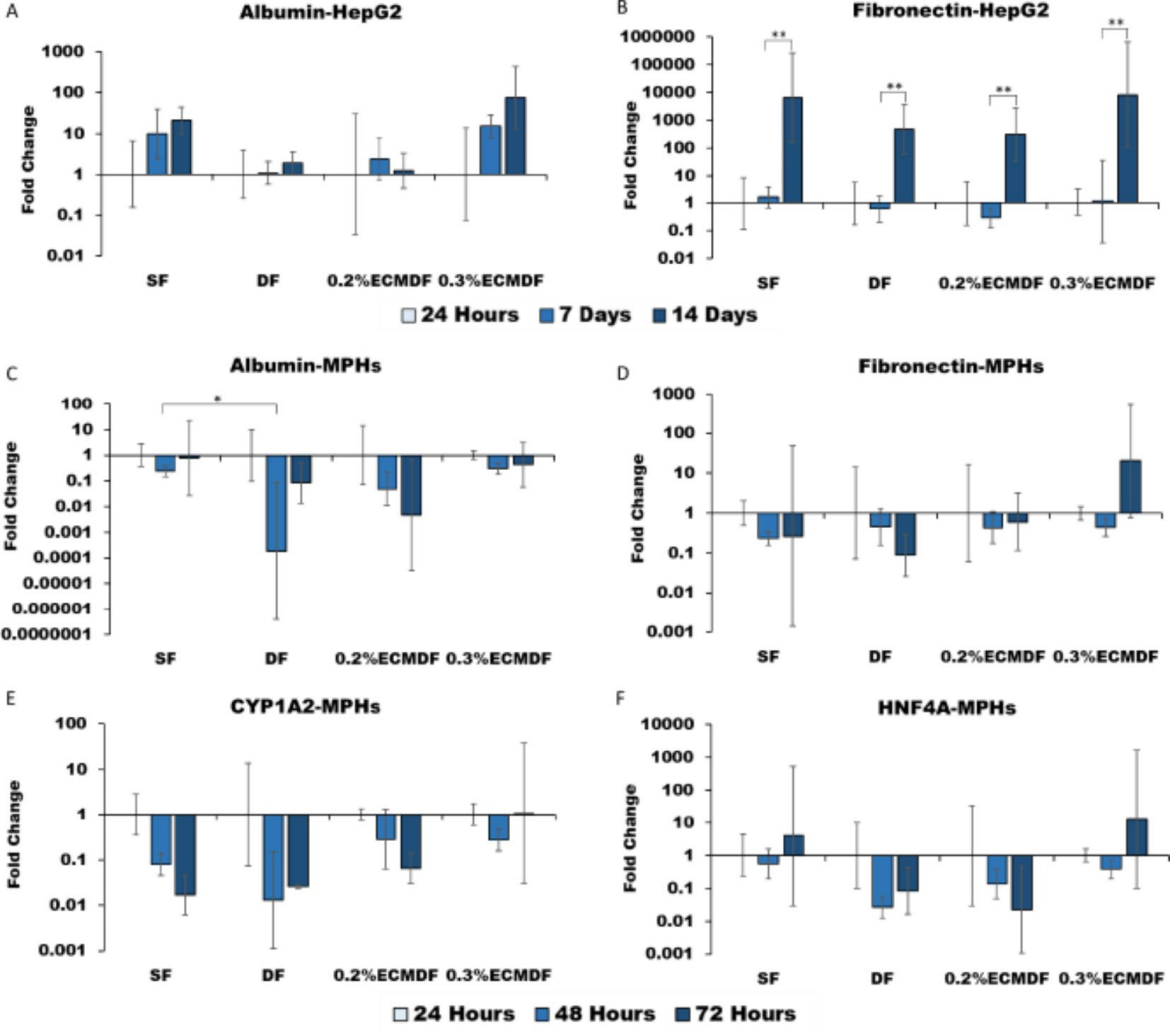
RT-qPCR gene expression of albumin and fibronectin produced by HepG2 and MPHs over culture period: (**A**) Albumin expression of HepG2; (**B**) Fibronectin expression of HepG2; (**C**) Albumin expression of MPHs; (**D**) Fibronectin expression of MPHs; (**E**) CYP1A2 expression of MPHs; (**F**) HNF4A expression of MPHs. All results were relative to the housekeeping gene GAPDH and normalised to each type of scaffold at 24 h. One-way ANOVA results with Tukey’s Post-hoc test shown. *= P value < 0.05. **= P value < 0.01. *N* = 5.

Q-PCR analysis obtained relative gene expression results for the hepatic function marker albumin and ECM gene fibronectin of HepG2 (Fig. 9A,B). Albumin expression, a marker of appropriate liver cell differentiation and function, was found to increase within scaffolds of HepG2 over 14 days, with the highest increase observed in 0.3% ECMDF at a 76.2-fold change. Only 0.2% ECMDF showed a slight decrease from day 7 to day 14. Fibronectin is known to contribute to ECM production in the liver, and is synthesised by primary hepatocytes in vitro^50^. HepG2 exhibited a significant increase from day 7 to day 14 (*P* < 0.01), with the sharpest increase observed in 0.3% ECMDF at an 8364.1-fold change.

MPHs were confirmed to show expression of albumin, fibronectin, CYP1A2 (Cytochrome P450 1A2) and HNF4A (hepatocyte nuclear factor-4 alpha) (Fig. 9C–F). On day 2 and day 3, albumin expression was down-regulated in all groups compared to day 1, and SF showed significantly higher expression than DF on day 2. Conversely, 0.3% ECMDF shows relatively stable expression with a 0.36-fold difference across three days. Likewise, the expression of fibronectin shows no real trend between the groups and time points. Interestingly, 0.3% ECMDF exhibits higher expression with a 10.8-fold upregulated on day 3, though no significant difference was observed between groups and time points. CYP1A2 and HNF4A were seen to decrease over the three days. However, on day 3, a recovery of CYP1A2 was observed in 0.3% ECMDF, and an up-regulation of HNF4A was seen in SF (3.9-fold) and 0.3% ECMDF (12.6-fold).

## Discussion

The present study illustrates the potential of using human liver tissue in electrospinning systems for the development of bioactive scaffolds for in vitro cell culture, which contains human-specific ECM proteins^51^. Human donor livers must fit strict criteria for transplantation, but on occasion can be deemed unsuitable for transplant due to various factors including poor liver graft quality or poor performance during ex situ machine perfusion or logistics^52^. In these cases, donor families may choose to allow research ensuring this valuable donation is given purpose regardless of suitability for transplant^53^. Our study provides an alternative approach for creating a bioactive environment for hepatocytes using human hepatic dECM, addressing the concerns of using animal tissue. In relation to the tissue used, no selection criteria were used for the donor. Donor availability was based on the liver being declined for transplantation. This tissue was declined because the clinical transplant team deemed it unsuitable for transplant (supplementary Fig. 1 for gross image).

Human liver tissue was successfully decellularized by agitation, allowing for the SDS solution to pass through the tissue. 0.1% SDS was chosen as it is known to effectively remove cellular components, whilst preserving the ECM proteins^54,55^. Quantification of the residual DNA showed an average 84% reduction from native tissue to decellularized samples. The amount of residual DNA ranged from 8.25 to 14.13 ng/mg, which is consistent with values reported in literature (13 ± 3.8 ng/mg), and significantly lower than the result obtained by Badylak (641 ng/mg) who used comparable detergent washing protocols^56,57^. A general approach of grinding dECM into powder is ball milling^10,12,58^, however we chose an alternative process, grinding dECM using a pestle and mortal, as well as homogenization followed by lyophilization^28^. Those methods have well performed in the previous studies^59^.

DMSO is a non-solvent for PCL, during the spinning process, as because of its high boiling point (189 °C), it will evaporate more quickly than CFM (61 °C)^60^. This causes the ratio of solvent/non-solvent to change and initiate phase separation. Depression structures form on the individual fibres when the fibre sheet is completely dry^33^. Water is highly miscible with DMSO but immiscible with CFM. Because of the high humidity in the spinning chamber, the water vapour condenses on the fibre surface and mixes with DMSO, eventually being adopted as a non-solvent, also resulting in depression structures^34^. However, NIPS is a more favourable mechanism in electrospinning since it can be controlled and the resulting sample is reproducible^61^.

When designing this manufacturing method, the key challenge was to maintain stable fibre morphology and surface topography whilst increasing the content of dECM. As known that the morphology of fibres affects cell behaviours, maintaining morphological consistency is essential in this study to elucidate the influence only from topography and dECM^27^. The commonly used spinning solvents for dECM are Hexafluoroisopropanol (HFIP) and 1,1,1,3,3,3-hexafluoro-2-propanol (HFP)^10,62^. However, in order to create the topography structure, a different solvent system has to be used. Previous studies have shown that organic solvents led to denaturation of various proteins, including collagen^63^. Chloroform can cause protein denaturation by disrupting non-covalent interactions between amino acids side chains, suggesting that the dECM used in this study could be denatured after electrospinning^64^. CFM is not a good solvent for dECM as high amounts of dECM cannot be dissolved and an unstable fibre morphology can be induced. This study reports an optimal dECM concentration (no larger than 0.3% w/w (dECM: PCL), see in supplementary material Fig. S1), which can simultaneously maintain the fibre morphology and topography while successfully exhibiting biological activity in the final products. As observed in the florescence of specific antibodies on the scaffolds and the differences in cell proliferation, morphology, and gene expression. Furthermore, the depression diameter (0.4 ± 0.15 μm) in this study was very similar to our previous study (0.37 ± 0.10 μm). Considering differences in polymer concentration, solution volume and additives (dECM), the final products exhibited relatively consistent topographic characteristics.

Immunostaining for collagen and fibronectin confirmed the presence of dECM within the scaffolds (Fig. 3). A similar approach was performed in our previous study demonstrating that small amounts of proteins or human/rat dECM (25 µg, 1 mg and 2 mg) can be efficiently incorporated in electrospun smooth fibres by direct blending, and influenced the key hepatic gene functions^10,28^. This suggests that the native protein structures, hence functions, may be not affected by decellularization, solubilisation and electrospinning progress, due to the specificity needed for immunodetection^65^. However, antibodies may occasionally bind to peptide fragments, thus the results do not fully confirm the specific binding of intact proteins^66^.

PCL has been used extensively as an electrospun scaffold material for tissue engineering, primarily due to its excellent biocompatibility, low toxicity and biodegradability^67^. While PCL is hydrophobic, which can limit cell adhesion and spreading. Our previous study has shown that the addition of a small amount of rat dECM improved the hydrophilicity of PCL electrospun scaffolds^28^. However, such a reduction in contact angle did not occur in the present study, this may be due to the different sources of ECM, the amount of incorporated dECM and spinning conditions^28^. Thus, we allude to a conclusion that although using similar methods to incorporate the dECM, the hydrophilicity is possibly affected by the tissue source and decellularization method.

The incorporation of dECM significantly influenced the mechanical properties of fibres. A reduction of Young’s modulus can be noted when increasing the amount of dECM, and the difference becomes smaller at higher strains (Fig. 3C). This impact has been demonstrated in previous studies^58,68^. This suggests that it will be possible to manipulate these variables to achieve a tissue-engineered construct with the desired mechanical properties. After degradation, DF shows a slight increase in weight loss, likely due to the porous nature increasing the specific surface area of the fibres, resulting in a larger contact area with media and a faster degradation rate. PCL hydrolytic degradation is sluggish due to its hydrophobic nature. Studies have shown no significant changes in weight loss of PCL electrospun scaffolds over extended periods of time, with degradation reaching only 1.44% after 90 days^69^. In terms of mechanical property, the overall value of Young’s modulus slightly increased (Fig. 4C) after degradation, which could be explained by the increase in the crystallinity of the material during degradation^69^. Furthermore, such results demonstrate that the mechanical influence of including the topographies and dECM on cellular behaviour in this study could have an impact on the biological results.

Increased cell metabolism and DNA content in HepG2 culturing over 14 days indicated high proliferative activity on the modified scaffolds compared to control bare PCL fibres (Fig. 5A,C). Whereas the fluorescence of the cell viability assay reduced on day 14, this could mean that the cells were in a lower metabolic state due to reaching high confluence as seen in Fig. 5. This finding is consistent with a similar observation reported by Bate et al^12^.. When adherent cells approach confluence and growth becomes contact inhibited, cell metabolism may slow down^70^. In the MPHs culture, both cell viability and DNA content decreased after day 1 (Fig. 5B,D), which is likely to be due to the known phenomenon of fragile MPHs dying off very quickly in vitro^71^. Additionally, the reduction in cell viability also could be due to various factors in the cell isolation, transportation, and seeding process. Cell damage occurs when cells are in a lower metabolic state, making them sensitive to stress and causing lower viability after 24 h of seeding^72^. However, no further decline was found between day 2 and day 3, which is a sign that MPHs maintained some bioactivity on these scaffolds after three days of culture. Primary hepatocytes are non-proliferative and quickly progress to apoptosis in vitro, which limits their culture period to a short duration^73,74^. Although some studies show longer culture duration, primary hepatocytes require specific growth factors and signalling molecules to maintain their viability and function. This study uses only basic media to provide an initial determination if these scaffolds can support primary cell survival and otherwise influence them. As a result, a three-day of culture of MPHs was performed. As stated in Fig. 5, the higher DNA content showed in hybrid scaffolds, with similar values compared to DF suggesting that the increase in bioactivity of all modified scaffolds is probably due to the increase in fibre surface area (depressions). Freshly isolated hepatocytes ideally predict liver toxicity in hepatic models and directly reflect the specific functionality of the liver. Ongoing research is investigating how to maintain the hepatic phenotype of primary hepatocytes in vitro and increase survival rate after transplantation in vivo^75^. There are still many remaining hurdles to overcome the transition from lab-based strategies to clinical feasibility^76^.

Albumin secretion was confirmed for both HepG2 and MPHs on all scaffolds (Fig. 6). Albumin is a key hepatic functional marker of hepatocyte function secreted by hepatocytes, and thus albumin production in vitro is essential for an effective model^77^. No major difference was observed between groups or time points, which is not supported by the increasing in HepG2 population or decreasing in MPHs. Therefore, the cell number may not be the driver behind the increase of albumin secretion, as it can also be seen in previous studies that different numbers of HepG2 cells produced a similar level of albumin during the culturing^43,44^. However, the concentration was augmented by 3D spheroid, Matrigel or collagen sandwich cultures^43,78,79^. This suggests that the nanotopographies and a small amount of additional biomolecules may not be the main drivers of albumin production.

Osmium-stained HepG2-seeded scaffolds show distinct cellular aggregates and morphologies (Fig. 7A,B). Both ECMDF exhibited a dense monolayer-like structure compared to the partially connected aggregates on DF and separated aggregates on SF on day 14. HepG2 also showed a better attachment on the modified scaffolds compared to SF. This observation has been demonstrated with other cells and polymers^80,81^. This can be in part explained by the presence of surface nanotopographies providing larger specific surface area which induces additional anchorage points for cells to attach, and thus improved the cell-material interactions. Identifying the optimal combination of scaffold parameters, including material, topography size, shape and direction, continues to present challenges in tissue engineering. This is primarily due to the complex pathways in combination with the time and costs associated with long-term in vitro and in vivo experiments. Silico predictive models could be a powerful tool to predict cell response to changes in topography and speeding up the design of optimal culture substrate^82^. Furthermore, the incorporation of dECM provides adhesion motifs such as Arg-Gly-Asp (RGD) (collagen, fibronectin) and Tyr-Ile-Gly-Ser-Arg (YIGSR) (laminin), which can improve the focal adhesion and monolayer-like structure formation as evidence previously showed in different types of cells and ECM proteins^36,37,62,83,84^. The non-significant cell morphology on MPHs suggests the influence of topography and dECM on MPHs are eligible for our systems.

The incorporation of depressions on the electrospun fibre did not significantly influence the albumin gene expression of HepG2 (Fig. 9A), which is consistent with previous work^18^. The observed increasing trend on 0.3% ECMDF however, can be considered due to the incorporation of dECM, as also observed previously^10,12,28^. For instance, it has been shown in previous studies that human dECM can alter the gene expression in liver cells of many key functional proteins^51,56^. This suggests that the incorporation of dECM within natural/synthetic polymers may become one of the most effective nanofibre scaffolds for promoting the functionality of HepG2. HepG2s are known to have low activity of expressing CYP genes so they were not assayed in this study^85^.

Fibronectin expression of MPHs showed downregulation over 3 days, whereas a recovery trend was found in both hybrid scaffolds, especially 0.3% ECMDF (Fig. 9D). The results suggest the incorporation of dECM might be the driving force for maintaining fibronectin expression, within the similar upward trends observed in the previous study^12^. CYP1A2 is a cytochrome P450 protein primarily associated with drug metabolism, was found to be influenced with the changes in morphology such as porosity of an electrospun scaffold^27^. Whereas, this study showed a largely unaffected expression of CYP1A2 on MPHs across the topographical changes (Fig. 9E). However, CYP1A2 is upregulated at day 3 on 0.3% ECMDF, indicating an improvement in metabolic capability for hepatocytes grown on the human dECM scaffolds, which is in line with the study by Grant et al.^10^. HNF4A acts as a transcription factor of mature hepatocytes specific genes, was reported as a key factor in maintaining hepatocyte-specific function in 3D culture system. No real trend was observed across scaffolds, but sharply increase was found on 0.3% ECMDF, and more than three times higher than SF on day 3 (Fig. 7F). Study previously showed that culture human primary hepatocytes in decellularized human liver scaffolds significantly increased the expression of HNF4A^86^. These results indicated these scaffolds retained MPHs phenotype in vitro, and 0.3% ECMDF has potential to improve the expression of functional marker (Albumin, CYP1A2 and HNF4A) and ECM gene (fibronectin).

The amount of dECM that can be blended was limited in order to maintain the consistency of fibre morphology. Another alternative way to immobilize dECM within biomaterials is carboxyl-to-amine cross-linking method using EDC (N-ethyl-N′-(3-dimethylaminopropyl) carbodiimide) / NHS (N-hydroxysuccinimide), which utilizes the crosslinking reaction between NHS-ester and free amino groups on ECM proteins^8,40^. In future studies, it may be possible to incorporate a higher amount of dECM into topography-modified fibres via chemical modification, though additional experimental procedures and chemicals are required for this. The challenge of using a human decellularized matrix includes variations in ECM composition between different donors. The ECM composition is highly dependent on environmental factors such as alcoholic liver and fibrotic liver, and some ECM proteins have been confirmed to have dynamic responses to stress changes^87^. Therefore, further investigation is needed to understand the effects of donor variability on cellular responses and to confirm the response can be related to the human dECM. Furthermore, we have some limitations as we didn’t include smooth fibre with dECM, we previously investigated rat and human dECM blended with smooth fibres^12,58,62^, and found that these biologically functionalized electrospun scaffolds supported cell growth and function. Therefore, we used smooth fibres as the control group in this study. However, in future studies, it would be considered to use dECM incorporated smooth fibre as one of the controls to get more insight into the observed effects due to dECM, topography, or their synergy. Additionally, the fibre size was constant at 2 μm as we need a stable platform to display our conditions, it’s worth acknowledging that previous work has shown liver cells respond to morphological changes (fibre size, orientation) in the electrospun scaffolds^27^. While these limitations are important considerations, they highlighted the potential of this approach to bioscaffold design that combines two key factors.

This work builds upon our foundation of knowledge that the role of human dECM as a complex biological cue to influence the responses of hepatocytes and its synergies with electrospun scaffolds in a lab-based environment, as well as its ability to be combined with nanotopographical signals for cells. This is the first time that topographically modified electrospun fibres have been combined with human liver dECM. Hepatoma cell lines and primary hepatocytes respond differently on these scaffolds. The optimal dECM concentration was determined in this study and the fibre morphology and topography remained consistent during the manufacturing process. Thus this novel hybrid scaffold has good reproducibility and has broad application prospects in different tissue engineering fields. This research provides an alternative approach to transforming human tissue, which is considered clinical waste, into valuable components for in vitro cell models and future therapeutics.

## Methodology

### Ethic approval and tissue storage

The use of human livers declined for transplantation was approved by the Lothian research and ethics committee (LREC, reference number 15/SS/2018), Lothian Research and Development (project number 2015/0408) and the NHS blood and transplant (NHSBT) Research Innovation and Novel Technologies Advisory Group (RINTAG; registered as study 56). Informed consent was obtained from the subject and/or their legal guardian(s) in accordance the Lothian research and ethics committee donor tissue usage. All methods were carried out in accordance with relevant guidelines and regulations approved by the Lothian research and ethics committee donor tissue usage. The obtained donor tissue was immediately sliced into 5 mm sections using a rotating blade apparatus (Host). The tissue slices were placed into separate polythene zip-lock bags and stored at -80 °C. Only one donor of human tissue (67 years, Male, Brain Stem Death) was used in this study.

All animal experiments were approved by the University of Edinburgh Animal Welfare and Ethical Review Body and the UK Home Office. All animal experiments were performed at the Centre for Regenerative Medicine, Edinburgh according to procedural guidelines and severity protocols from the UK Home Office Animals Scientific Procedures Act.

### Decellularization

Decellularization was performed using previous methods, following optimisation^56^. A slice of human liver tissue was defrosted at room temperature (RT) and chopped into cubes smaller than 5 cm^3^ by a knife, and placed in glass beakers agitated on a Pro 30 Reciprocal Shaker (Labnet) at 125 rpm at RT. Samples were first incubated in 200 mL PBS for 4 h. Subsequently, the tissue was transferred into 200 mL of 0.1% sodium dodecyl sulphate (SDS, Sigma) in dionised water and incubated for 12 h. On day 2, the solution was replaced with fresh 0.1%SDS for 4 h of incubation, then replaced again with fresh 0.1% SDS and incubated for 12 h. On day 3, the solution was exchanged with 200 mL Milli-Q water and kept for 4 h, followed by another exchange and incubated for 12 h. On day 4, the steps on day 3 were repeated. On day 5, the tissue was washed twice with 200 mL Milli-Q water for 1 h each to ensure complete detergent removal. The decellularized tissue was collected and ground with a glass pestle and mortar. The solution obtained was then lyophilized overnight in a FreeZone 4.5 freeze-drier (Labconco) and stored at -80 °C until use.

### ECM processing

30 mg decellularized tissue was digested with 1 mg/mL pepsin (Sigma, 3706 units/mg) in 3 mL 0.01 M Hydrochloric acid (HCL, Sigma) in Milli-Q water (dECM concentration is 10 mg/mL) with agitation on a SRT9D roller mixer (Stuart) at RT for 72 h. The digested solution was neutralized with 0.1 M Sodium Hydroxide (NaOH, Sigma) to a final pH of 7 to stop the digestion. Then, the solution was lyophilized and stored at 4 °C until further use.

### Electrospinning for cell-seeded scaffolds

Electrospinning solutions for each type of fibre were prepared as described in Table 2. Briefly, 2 mg or 3 mg dECM was added to 2 mL chloroform (CFM, Sigma) and manually ground to obtain a suspension solution in a hood using a 3 mL borosilicate glass tissue homogenizer. This solution was then mixed with an additional 3.4 mL CFM and 0.6 mL DMSO (Sigma) in a glass vial. 1 g polycaprolactone (PCL) pellets (Mn = 80 000 Da, Sigma) were added and incubated with agitation on the roller mixer at 8 rpm overnight. The depression fibres (DF) were fabricated using the above method without the addition of dECM. The smooth fibres (SF) were made by dissolving 1 g PCL in 5 mL CFM and 1 mL Methanol (MeOH, Sigma). All scaffolds were fabricated at RT (∼20 °C) with a relative humidity of ∼55% using the following parameters, voltage 13 kV/-4 kV, flow rate 2.5 mL/h, mandrel-to-needle distance 23 cm, mandrel speed 250 rpm, needle diameter 0.8 mm, and the working distance (sheet width) 60 mm. The fibres were collected on aluminium foil; the obtained fibre sheets were dried in a hood overnight and stored at 4 °C until use.

**Table 2:** Electrospinning solution parameters.

	Smooth fibre (SF)	Depression fibre (DF)	0.2% ECM-depression fibre (0.2%ECMDF)	0.3% ECM-depression fibre (0.3%ECMDF)
*PCL content*	1 g	1 g	1 g	1 g
*CFM*	5 mL	5.4 mL	5.4 mL	5.4 mL
*DMSO*	-	0.6 mL	0.6mL	0.6 mL
*MeOH*	1 mL	-	-	-
*ECM content*	-	-	2 mg	3 mg

### Scanning electron microscopy of scaffolds

Acellular scaffolds were sputter coated with gold-palladium using an Emscope SC500A splutter coater to increase electrical conductivity. Coated samples were visualized using a SEM (Hitachi TM4000) at 15 kV acceleration voltage in mixed sensor mode. Small magnification (x3000) was used to calculate the fibre/depression diameters (50 measurements were taken for each sample); large magnification (x10000) was used to visualize the topography of fibres. All images were processed and measurements were performed manually using ImageJ software.

### Contact angle measurements

A DMK 41AU02 (Imaging-Source) monochrome 1,280 × 960 camera was used to measure the water contact angle of dry scaffolds. A 5 µL water droplet was pipetted onto each scaffold and images were captured every 0.02 s at 5 Hz. The images at 0 to 1 s were chosen, and measurements were performed in ImageJ software with the contact angle plugin.

### Mechanical testing

An Instron 3367 tensile testing machine (Instron) was used to measure the mechanical properties of the scaffolds, as previously described^18^. Briefly, samples were cut into rectangular strips with a 20 mm gauge length and 5 mm width using a scalpel. Thickness was measured using the DMK 41AU02 camera and calculated using ImageJ software. Samples were fixed to a “C” shape paper to ensure a consistent gauge length to improve handling ability. All samples were subjected to 50 N monotonic tensile loading at a strain rate of 50% strain per minute until failure. The ultimate tensile strength and Young’s modulus were calculated, and the incremental Young’s modulus was taken at a range of strain percentiles: 0–5, 5–15, 15–25, and 25–35%.

### Electrospun scaffolds for evaluation of in vitro degradation

Electrospun scaffolds were manufactured using comparable methods to the cell-seeded scaffolds. The electrospun fibres were cut into rectangular strips with a 20 mm gauge length and 5 mm width, as described above. These samples were weighed for the initial dry weight (Wo). Samples were sterilized with 70% ethanol for 15 min, followed by 30 min in a fresh 70% ethanol solution in a bio hood. Subsequently, samples were washed with sterilized PBS three times for 10 min each, and part of the samples were collected and dried 24 h in the fume hood as “Before degradation” scaffolds. The rest of the samples were incubated in 5 mL serum-free Eagle’s minimum essential medium (MEM, Gibco) containing 1% antibiotic/antimycotic (Anti-Anti, Gibco) at 37 °C. After 14 days, the samples were removed from the media and washed in deionised water for three times ten minutes each, and dried in the fume hood for 24 h as “After degradation” samples. The degraded scaffolds were weighed again as the dry weight after degradation (W_d_). Weight loss as a percentage of the initial dry weight was calculated according to Eq. (1). The physical appearance of the samples after degradation was examined by SEM (JEOL JSM-IT100, JEOL Ltd., Japan) and analysed in ImageJ software. The mechanical properties of the samples after degradation were quantified according to the methods previously described.

1 $$\:Weight\:loss\:\%=\frac{Wo-Wd}{Wd}\times\:100\%$$

### Scaffold preparation

Scaffold discs were punched out using a 10 mm biopsy punch, and immersed into 70% ethanol (Sigma) for 15 min to remove the aluminium foil backing. Then samples were sterilized in 70% ethanol for a further 30 min and allowed to dry completely. The samples were moved into a bio hood and washed with sterilized PBS three times for 10 min each. They were incubated (5% CO_2_ and 37 °C) in serum-free media (MEM, Gibco)) containing 1% Anti-Anti overnight before seeding.

### Immortalised cell line culture and seeding

HepG2 cells (Sigma) at passage 9 were cultured in complete media containing MEM (Gibco) supplemented with 1% L-glutamine (Gibco), 1% Non-essential Amino Acids (NEAA, Sigma), 1% Penicillin–Streptomycin (Gibco), and 10% foetal bovine serum (FBS, GE Healthcare) for 7 days to reach 70% confluency. Cells were trypsinised by a 5 min incubation in 3 mL Trypsin-Ethylenediaminetetraacetic acid (EDTA) (Sigma), and resuspended in 1.5 mL complete media. 20 µL of the cell suspension (cell density 1 × 10^4^) was seeded directly onto each scaffold. The seeded scaffolds were then incubated at 37 °C and 5% CO_2_ for 1 h before 30 µL complete media was added to avoid drying. After an additional hour to allow cell attachment, 500 µL of complete media was added to each scaffold. Samples were collected at 24 h, 7 days and 14 days; the media was changed every three days.

### Mouse primary hepatocytes isolation and seeding

Mouse primary hepatocytes (MPHs) were obtained by following a standard two-step collagenase perfusion technique^88^. Briefly, eight-week-old male C57BL/6J mice (Charles River) were injected with a terminal dose of anaesthetic (200 mg/kg Ketamine, Chanelle Pharma; 3 mg/kg Medetomidine Orion Pharma), followed by a midline laparotomy through the skin and muscle layers to uncover the liver and locate the hepatic portal vein. The hepatic portal vein was cannulated and perfused with 50 mL pre-warmed (37 °C) liver perfusion media, followed by 50 mL liver digest media (both Gibco). The liver was removed and hepatocytes were mechanically disassociated and released from the Glisson capsule. The solution was filtered through a 70 µM filter and purified by density gradient centrifugation. All cells were resuspended in 5 mL attaching media (Dulbecco’s Modified Eagle’s Medium (Sigma) supplemented with 10%fetal calf serum (FCS), 23% Ham’s Nutrient Mixture F12 (Sigma), 0.6% Bovine Albumin Fraction V Solution (Gibco), 50 µL L-proline (Sigma), 50 µL EGF (Murine, Peprotech), 1% Insulin-Transferrin-Selenium X (Gibco), 5 µL Dexamethasone (Sigma), 0.1% Nicotinamide (Sigma), 0.06% 2-Phospho-L-ascorbic acid trisodium salt (Sigma), 1% Penicillin/Streptomycin (Invitrogen), 1% L-glutamine (Invitrogen), 0.5% HEPES Sodium Salt (Gibco)). 20 µL cell suspension (cell density 1.2 × 10^5^) was seeded directly onto each scaffold and incubated for one hour to allow cells to attach. 30 µL attaching media was subsequently added to each sample to avoid drying. After 1.5 h, 500 µL culture media (attaching media without FCS) was added to each well. Samples were collected at 24 h, 48 h and 72 h; the media was changed every day. The use of animals in this study is reported in accordance with ARRIVE guidelines where applicable (https://arriveguidelines.org).

### Cell viability assay

The CellTiter-Blue assay (Promega) was used to determine cell metabolic activity, according to the manufacturer’s protocol. Briefly, cell-seeded scaffolds were moved to a new 24-well plate. 500 µL of CellTiter-Blue reagent and cell culture media (ratio 1:4) were added to each well. After 3.5 h of incubation at 37 °C and 5% CO_2_, 100 µL solution from each well was added to a 96-well black microplate. Measurements were performed using a CLARIO-star Plus microplate reader (BMG LABTECH) at the excitation wavelength of 525 nm and emission wavelength of 580–640 nm.

### DNA quantification

The Quant-IT Picogreen dsDNA assay (Promega) was performed to quantify the DNA content of cell-seeded scaffolds and determine the effectiveness of liver tissue decellularization. Native and decellularized tissue, as well as cell-seeded scaffolds were enzymatically digested with papain solution containing 2.5 U/mL of papain, 5 mM cysteine-hydrochloride (HCL) and 5 mM EDTA in DNA-free water (all Sigma). Digestion was performed for 48 h and 24 h for tissues and scaffolds respectively, in an Eppendorf ThermoMixer C (65 °C, 450 rpm). The aqueous working solution was prepared according to the manufacturer’s protocol and added with the digested solution into a 96-well black microplate to achieve a total volume of 200 µL. A ladder of 0, 1, 10, 100 and 1000 ng/mL of dsDNA was prepared for standard solution to calibrate fluorescence intensity versus dsDNA concentration. Measurements were performed using a CLARIO-star Plus microplate reader at the excitation wavelength of 480 nm and emission wavelength of 510–570 nm.

### Albumin secretion

Albumin concentration in media samples was conducted using the Bromocresol green (BCG) assay reagent (Sigma), according to the manufacturer’s instructions. 24 h before media collection, the media on the cultures was changed to ensure a 24-hour secretion measurement. Media was then collected at each time point (24 h, 7 days, and 14 days) and stored at -80 °C. 5 µL of sample was added to 100 µL of BCG reagent and incubated at room temperature for 5 min in a 96-well clear bottom plate. The absorbance at 620 nm was then measured for all samples alongside a ladder of standard solutions using CLARIO-star Plus microplate reader. Absorbance values were correlated with a standard curve to quantify albumin content within the media.

### Histology staining

Native and decellularized liver tissues were fixed in 10% Formalin (Sigma) overnight at 4 °C. This was followed by a series of ethanol (50–100%) dehydration. Samples were washed in xylene (Sigma) before being embedded in paraffin wax. The embedded samples were trimmed to 10 μm thickness and mounted onto slices for haematoxylin and eosin (H&E) and Picrosirius Red staining. Samples were cleared with xylene and mounted in ProLong Gold Antifade Reagent (Invitrogen) after staining. Images were taken with Nikon TE2000 Inverted and processed using icy software.

### Fluorescent staining

The samples for fluorescent staining were collected at each time point and placed in 10% formalin for 30 min at room temperature. Samples were then transferred to PBS and stored at 4 °C until use. Formalin-fixed samples were permeablized with 0.2% TritonX-100 (Sigma) in PBS for 10 min. After three PBS washes, each sample was stained with 1µL/mL fluorescent conjugated Phalloidin 514 (Sigma) in 1% Bovine serum albumin (BSA, Sigma) in PBS for 30 min, followed by three PBS washes. Samples were stained with 300 nM 40,6-diamidino-2-phenylindole (DAPI, Thermo-Fischer) in PBS for 15 min followed by three PBS washes. Cells were visualized and imaged with a Zeiss Axio Imager fluorescent microscope and processed using icy software.

### Immunohistochemistry staining

In order to determine the presence of ECM proteins throughout the acellular scaffolds, samples were stained with fluorescently conjugated collagen I and fibronectin according to the manufacturer’s protocol (Abcam). Briefly, acellular scaffolds were blocked with 1% BSA in 2.3.in PBST overnight at 4 °C. Samples were washed three times with PBS, and stained with 1µL/mL fluorescently conjugated rabbit IgG secondary antibodies (Abcam) for 1 h at room temperature. After three PBS washes, stained samples were stored at 4 °C and then visualized with Zeiss Axio Imager fluorescent microscope.

### Osmium treatment and SEM of cell-seeded scaffolds

Cell-seeded scaffolds were visualized using SEM using a previously described osmium-based method^89^. Briefly, the samples were collected at each time point and placed in 4% glutaraldehyde (Sigma) for 30 min at RT. Samples were then transferred to PBS and stored at 4 °C until use. Glutaraldehyde fixed scaffolds were placed in 0.1% osmium tetroxide (TAAB) in deionised water for 30 min. Scaffolds were dehydrated in a series of standard ethanol dilutions (30–100%) and hexamethyldisilazane (HDMS, Sigma) for 30 s each. Samples were left in fresh HDMS and dried overnight in the fume hood. All images were captured using Hitachi SEM and processed by ImageJ software. Images for each group were taken at the same magnification (x150) and were thresholded to distinguish cells and fibres (background), and the percentage of surface area covered by cells was calculated to determine the percentage of confluence.

### Reverse transcription polymerase chain reaction (RT-PCR)

The samples for gene expression analysis were put into 500 µL Tri-Reagent (Invitrogen, Thermo-fisher) and stored at -80 °C until use. RNA was extracted from thawed samples by adding 100 µL CFM and centrifuged at 12000 g for 10 min. The aqueous RNA supernatant was taken out and placed into 200 µl 70% ethanol. The RNA/ethanol solution was purified using Qiagen’s RNeasy spin column system according to the manufacturer’s instruction, and eluated with 20 µL Nuclease-Free water (Invitrogen). A nanodrop spectrophotometer (Thermo) was used to determine the nucleic acid concentration. The RNA concentration was equalised by adding Nuclease-Free water. Then RNA was reversed-transcribed to complementary DNA (cDNA) using the Improm-II Reverse Transcription kit (Promega) according to the manufacturer’s instructions. cDNA samples were stored at -20 °C until use. Quantitative real-time polymerase chain reaction (qRT-PCR) was conducted using the LightCycler 480 Instrument II (Roche Life Science) and the Go-Taq qPCR system (Promega). The results were relative to the housekeeping gene Glyceraldehyde 3-phosphate dehydrogenase (GAPDH) and normalized to each condition at 24 h). Analysis was performed using the 2^−∆∆*Ct*^ method.

### Statistical analysis

Statistical analysis was performed using a One-way ANOVA and Tukey’s post-hoc test in Minitab 18 software, 3 ≤ *N* ≤ 5. P-values below 0.05 were considered statistically significant and presented in figures as * *p* ≤ 0.05, ** *p* ≤ 0.01 and *** *p* ≤ 0.001. Error bars represent standard deviation (SD), and all results are expressed as mean ± SD.

## Electronic supplementary material

Below is the link to the electronic supplementary material.

Supplementary Material 1

## Data Availability

All data generated or analysed during this study are included in this published article.
