# Supplementary material for: Combining human liver ECM with topographically featured electrospun scaffolds for engineering hepatic microenvironment
Source: Sci Rep. 2024 Oct 5;14:23192. doi: 10.1038/s41598-024-73827-5 (PMC11455933; doi:10.1038/s41598-024-73827-5)
Supplement: Supplementary file 1 — Supplementary Material 1 [file 41598_2024_73827_MOESM1_ESM.docx]

**Supplementary Materials**


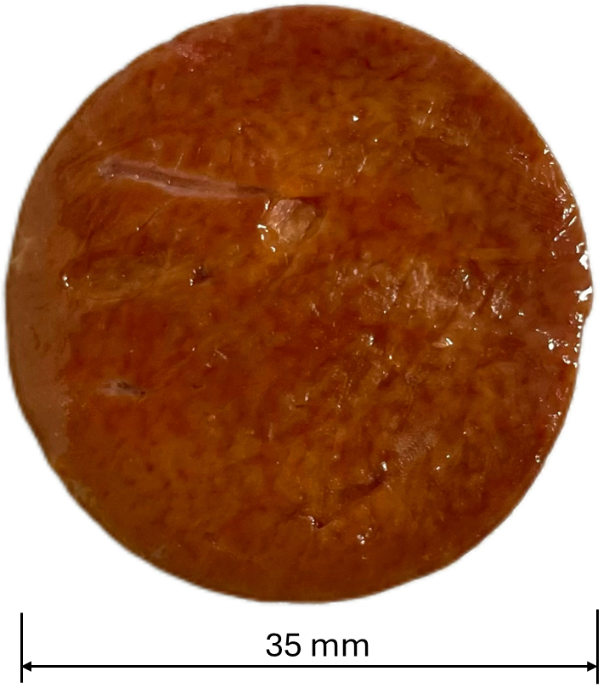


Supplementary Figure 1. A disc image of tissue from one donor was used in this study.

**
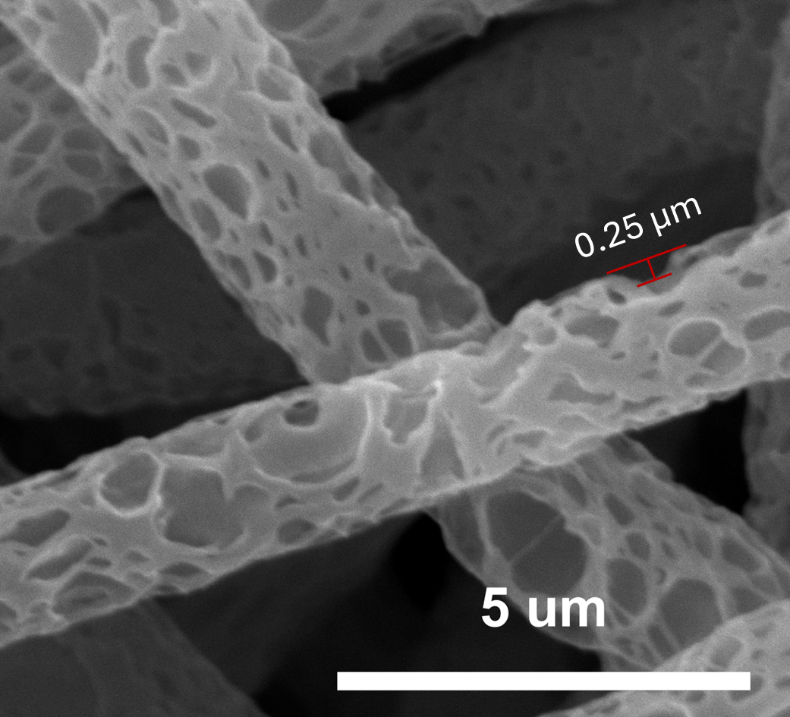
**

Supplementary Figure 2. SEM images of DFS scaffolds show the details in size on the depression surface.
